# Cerebellar Cognitive Affective Syndrome: A Two‐Case Report

**DOI:** 10.1002/alz70857_104443

**Published:** 2025-12-25

**Authors:** Marcelo Jobet, Veronica Perez, Rodrigo A Santibanez, Patricio Mellado

**Affiliations:** ^1^ Memory Clinic, Neurology Service, Complejo Asistencial Doctor Sótero del Río, Santiago, Chile; ^2^ Neurology Department, Faculty of Medicine, Pontificia Universidad Católica de Chile, Santiago, Chile; ^3^ Pontificia Universidad Catolica de Chile, Santiago, Chile

## Abstract

**Background:**

Cerebellar cognitive affective syndrome (CCAS) is clinically characterized by impaired functioning across all cognitive domains, particularly executive function and mood regulation. CCAS is attributed to lesions compromising cerebellar structures that modulate cognition and affection. Diagnosis is based on identifying neuropsychiatric symptoms in patients with cerebellar lesions, supported by neuropsychological assessments, especially the CCAS scale (CCAS‐S). We report two patients diagnosed with CCAS evaluated in our clinic in 2024.

**Method:**

The first case, a 72‐year‐old man, presented with progressive cognitive complaints and personality changes 3 years after a cerebellar stroke. He had become less communicative and reported difficulties planning, organizing, and performing tasks he previously did effortlessly. His family also noted forgetfulness and working memory problems. He scored 15/30 on the Montreal Cognitive Assessment (MoCA). Brain MRI did not feature brain atrophy; however, it showed multiple chronic infarcts in the cerebellum, mesencephalon, and pons. He underwent neurocognitive evaluation where he underscored in all cognitive domains, particularly in attention, executive, and visuospatial functions. In the CCAS‐S he failed 8/10 items with a raw score of 50/120. The second case is a 65‐year‐old man with a history of infarcts affecting multiple cerebellar territories, who clinically presented with diminished verbal fluency and deterioration of executive functioning, with no significant personality changes or memory loss. He had no evidence of brain atrophy in structural neuroimaging and scored 28/30 in MoCA. He failed 5/10 items and had a raw score of 65/120 in the CCAS‐S. They were both diagnosed with CCAS.

**Result:**

Both patients lacked evidence of a neurodegenerative process or supratentorial lesion, and both had sequelae of lesions involving the cerebellum. The clinical suspicion of CCAS was reaffirmed with the CCA‐S.

**Conclusion:**

CCAS is a cause of cognitive and behavioral impairment different from classic neurodegenerative pathologies. High clinical suspicion, exclusion of alternative causes, and neurocognitive assessment are needed to achieve accurate diagnosis. The CCAS‐S appears to be a useful tool to assess suspected CCAS cases. Further research is needed to clarify the physiopathology of CCAS and improve its diagnosis and treatment.

